# Determining psychometric properties of the Persian version of demoralization scale-II in patients with cancer

**DOI:** 10.1186/s40359-023-01507-6

**Published:** 2024-01-02

**Authors:** Elaheh Taghilou, Mehdi Heidarzadeh, Behnam Molaei, Mehdi Ajri Khameslou

**Affiliations:** 1 School of Nursing and Midwifery, Ardabil University of Medical Sciences, Ardabil, Iran; 2 School of Medicine, Fatemi Hospital, Ardabil University of Medical Sciences, Ardabil, Iran

**Keywords:** Cancer, Demoralization, Depression

## Abstract

**Background:**

One of the emotional problems in patients experiencing cancer is demoralization syndrome. Concerning the importance of demoralization in patients with cancer, having a valid and reliable scale for assessing this problem is crucial. A revised version of Demoralization Scale (DS-II) was designed in 2016. It was necessary to determine its validity and reliability in populations with different cultures. This study aimed to determine the psychometric properties of the Persian version of DS-II (PDS-II) in Iranian patients with cancer.

**Methods:**

The study population comprised 170 Iranian patients with cancer in Ardabil, Iran. The inclusion criteria were: age 18 or more, ability to understand and speak Persian, willingness to consent to participate in the study, having healthy cognitive function, and having an awareness of cancer. To determine the psychometric properties of PDS-II, the content, convergent, construct, and discriminant validity, besides internal consistency, were evaluated.

**Results:**

Regarding the high correlation of PDS-II with General Anxiety Disorder, Patient Health Questionnaire, Beck Hopelessness Scale, and Hospital Anxiety and Depression Scale, the convergent validity of the PDS-II was confirmed. Confirmatory factor analyses confirmed both the original 2-factor and one-factor models of PDS-II. Internal discriminant validity of the PDS-II was not confirmed because the Average Variance Extracted from two dimensions of PDS-II (AVE = 0.31 and 0.37) was less than the square correlation between these two dimensions (r^2^ = 0.79). Cronbach α and coefficient was 0.88 for the PDS-II.

**Conclusions:**

PDS-II is a valid and reliable scale for measuring demoralization among Iranian people with cancer. However, the discriminant validity of the PDS-II was not supported.

## Background

Cancer incidence and mortality rates were estimated at 19.3 million new cases of cancer and almost 10.0 million deaths from cancer worldwide in 2020 [1]. The number of new cancer cases in Iran was 131,191 in 2020. This year, stomach, prostate, lung, colorectal, and bladder were the most common cancers in males, and in females, the most common cancers included: breast, colorectal stomach, lung, and thyroid [2].

Patients with cancer often experience many physical, psychological, and social problems, such as fatigue, sleep disturbances, pain, nausea, diarrhea, neuropathy, skin rashes impaired sexual functioning, anxiety, depression, worry, fear, separation from family, relationship problems and social isolation that affect many life aspects of the patients and their families [3–7].

One of the emotional problems in patients experiencing cancer is demoralization syndrome. Demoralization is a maladaptive coping characterized by a loss of purpose and meaning in life, low morale, low optimism, helplessness, and hopelessness [8]. Demoralization can influence mood and the ability to cope with threatening life events; it also negatively impacts social functioning, decision-making, and quality of life [9] and causes a feeling of dependence and a burden on others [8, 10]. This concept provided an essential and profound basis for evaluating the existential distress in patients with cancer not treated by standard diagnostic approaches [11]. Robinson et al. stated that demoralization syndrome is common (13-18%) in patients with progressive diseases [8]. Concerning the importance of demoralization in patients with cancer, having a valid and reliable scale for assessing this problem is crucial.

There are some instruments designed to measure demoralization. The Demoralization Scale (DS) was initially validated in 2004 to measure demoralization in patients with advanced cancer [12]. It was originally validated among patients with advanced cancer and consists of 5 subscales. Because of some limitations such as the length of the scale, some reversed items, and poor discriminant validity of DS, the designers of the scale changed the original 24-item version of the DS to an easier-to-fill-in, 16-item version which measures demoralization through 2 factors: Meaning and Purpose, and Distress and Coping Ability [13].

Some studies have assessed and confirmed the validity and reliability of the Demoralization Scale II (DS-II) in different samples [14–16]. Nevertheless, there are some ambiguities in the factor structure of DS-II in the mentioned studies. For example, while Robinson et al. (2016) introduced DS-II as a bifactor scale [16], Koranyi et al. (2021) showed that the one-factor DS-II model is as fit as the two-factor model [15]. On the other hand, this tool has not been used in Iranian society. Previous studies have shown that chronic patients in Iranian society show different adaptive behaviors in critical situations [17], so it is necessary to determine the validity and reliability of the DS-II in the Iranian population. One of the main critiques of 24-item DS was its poor discriminant validity, but this importance has not been investigated in the new 16-item DS in previous studies. This study aimed to determine the psychometric properties of DS-II in Iranian patients with cancer with special analyses of the factor structure and discriminant validity of DS-II.

## Methods

This methodological study translated the DS-II and validated its Persian version among Iranian patients with cancer.

### Participants

The study population comprised Iranian patients with cancer referred to the oncology department and oncology clinic of Imam Khomeini Hospital of Ardabil University of Medical Sciences, Ardabil, Iran. The convenience sampling method was used according to the inclusion criteria for selecting participants. The inclusion criteria were having cancer according to an oncologist confirmation, age 18 or more, ability to understand and speak Persian, willingness to consent to participate in the study, having healthy cognitive function based on history and clinical records, and having an awareness of cancer. The exclusion criteria were psychological and/or cognitive diseases (according to the patient’s history and medical records), inability to answer the questions, and unwillingness to continue answering questions. Filling the questionnaires and/or interviewing with them were performed at patients’ bedside. For this, sufficient time was allocated for each interview and the interviews were arranged for a time when the participants were ready to do it.

#### Sampling and sample size

A convenience sampling method was used to select the eligible samples. 235 patients with cancer were admitted to the clinics over 6 months (from August 2022 to January 2023); however, 52 of these patients did not have the inclusion criteria (32 people could not communicate in Farsi, 15 people were not aware of their diagnosis, and 5 were less than 18 years old). Eventually, of the 183 remaining patients, 170 consented to participate in the study. Considering that 5 to 20 samples per item were required to perform the confirmatory factor analyses [18], a minimum of 80 samples was considered sufficient to achieve this goal. On the other hand, considering α (two-tailed) = 0.05, β = 0.20, and the correlation coefficient of 0.36–0.72 obtained in previous studies [14, 15], 13–79 samples were needed to perform convergent validity. As the estimated sample size could be used for all purposes of the study, therefore, the information obtained from 170 participants was considered acceptable to achieve the objectives of the study.

### Data collection

#### Instruments

##### Demoralization scale II (DS-II)

The DS-II is a self-report questionnaire with 16 items rated using a three-point Likert scale (0 = “never”; 1 = “sometimes”; 2 = “often”), resulting in a total score ranging from 0 to 32. The DS-II contains two 8-item subscales: meaning and purpose and distress and coping ability. High scores represent high levels of demoralization [16].

##### Patient health questionnaire (PHQ-9)

The PHQ-9 is a self-report measure designed to assess the presence and severity of a major depressive episode (MDE) and is comprised of 9 items [19]. Items are rated on a 4-point Likert scale ranging from 0 (not at all) to 3 (nearly every day) concerning whether the symptom has been experienced in the past 2 weeks. The sum score ranges from 0 to 27, and higher scores represent higher depressive symptoms. The Persian version of PHQ-9 shows good internal consistency [20].

##### Beck hopelessness scale (BHS)

The BHS comprises 20 dichotomous “true/false” items to assess three major aspects of hopelessness: feelings about the future, loss of motivation, and expectations. Higher total scores indicate greater hopelessness (range 0–20) [21]. The Persian version of the BHS has been translated and validated by Kaviani et al. (2001) [22].

##### General anxiety disorder (GAD-7)

The GAD-7 is a 7-item questionnaire developed to identify probable cases of GAD and measure the severity of GAD symptoms [23]. The GAD-7 assesses the most prominent diagnostic features (diagnostic criteria A, B, and C from the Diagnostic and Statistical Manual of Mental Disorders, fourth edition [DSM-IV]) for GAD [23]. The Persian version of the GAD-7 has been translated and validated by Nainian et al. (2011) [24].

##### Hospital anxiety and depression scale (HADS)

The HADS contains 14 items and consists of two subscales: anxiety and depression. Each item is rated on a four-point scale, giving maximum scores of 21 for anxiety and depression. Scores of 11 or more on either subscale are considered a significant ‘case’ of psychological morbidity, while 8–10 represent ‘borderline’ and 0–7 ‘normal’ [25]. The Persian version of the HADS has been translated and validated by Kaviani H et al. (2009) [26].

##### Translation process

After getting permission from the main developer of the scale, other steps of the study were begun. The translation and validation of the DS-II were performed according to the method suggested by Wild et al. (2005) [27]. The DS-II was translated into Persian by two people fluent in English and Persian. Furthermore, the two translations were compared and edited, and a final copy was prepared. Then, the final version was translated back into English by another person fluent in Persian and English. Moreover, two versions of DS-II (the back translation and original scale) were assessed by a supervisor, and a few minor revisions were carried out in the Persian version. There was no difference between DS-II and the Persian version of DS-II (PDS-II) in the number and content of items. After approval of the final version of PDS-II, the validation process was carried out using content validity, construct validity, and internal consistency reliability.

##### Content validity

The Content Validity Index (CVI) was calculated to determine the content validity. To assess CVI, 4 oncology nurses and 8 nursing faculty members were asked to comment on the translated scale. These people were chosen based on their experience handling patients with cancer or working on instrument development. These experts scored each item of DS-II, in a range of 1 to 4, for relevance, simplicity, and clarity. CVI for each item was calculated as the number of experts who gave a rating of either 3 or 4 divided by the total number of experts; CVI in the whole scale was calculated as the proportion of items on the scale that achieved a rating of 3 or 4 divided by all the content experts [28]. Based on the views of all 12 experts, the CVI of the whole scale was calculated as 0.94. The results of content validity confirmed all 16 items in the form of two dimensions.

##### Convergent validity

To check the convergent validity of the PDS-II, the GAD-7, PHQ-9, BHS, and HADS were simultaneously completed by the participants and their correlation with the PDS-II was calculated. It was assumed that in the case of convergent validity, there is a positive relationship between PDS-II and the scores obtained from these scales.

##### Construct validity

The construct validity of the PDS-II was initially assessed using indices of Confirmatory Factor Analyses (CFA) and standardized coefficient, followed by further assessment using the discriminant validity by the structural equation model (SEM). It was supposed that the factors in the scale were so distinct despite their correlations that no two factors could be correlated as one or the same factor. For this purpose, two stages were used: First, unidimensionality and multidimensionality of PDS-II were determined. It was assumed that the multidimensional model should be considered if the unidimensional model did not fit [29, 30]. So, in this step, two models were assessed. In the first model, we considered PDS-II as a two-factor model in which 16 items are completely similar to the original version (model 1). In the second model, 16 items of PDS-II were evaluated as a one-factor model (Model II). Second, The Average Variance Extracted (AVE) was compared with the square of the correlation between factors. The evidence for discriminant validity is known to be provided when the square of correlations between factors is less than every single AVE [30].

We also examined the discriminant validity of the PDS-II by using the items of PHQ through structural equation modeling (SEM). For this purpose, 16 items of PDS-II were considered one factor, and 9 items of PHQ-9 were considered the second factor (model III). We aimed to calculate the AVE in each factor and the square of the correlation between factors. It was assumed that in the case of discriminant validity of the PDS-II, the AVE of factors (PDS-II and PHQ in model III) should be greater than the square of correlation between these two factors.

##### External discriminant validity

To check the external discriminant validity, at first, the discriminant validity between major depression and demoralization was examined. Patients were divided into two groups with and without Major Depression Episodes (MDE). For this purpose, the criterion of major depression in the PHQ ≥ 10 was used to identify depression. So the patients were divided into two groups: patients who did not meet the diagnostic criteria for an MDE (PHQ score less than 10) and those who met the diagnostic criteria for an MDE (PHQ score equals 10 or more). Also, patients were divided into 3 low, moderate, and high demoralization groups according to PDS-II scores (low scorers, 0-25th percentile; middle scorers, 25th -75th percentile; and high scorers,75th percentile). Then cross-tabulation frequencies of PHQ and DS were examined in these groups. Also, the performance status of the participants was calculated using Karnofskey’s performance test. For this purpose, participants were divided into 3 groups: group 1 as normal activity (Karnofsky Performance Score: 80–100), group 2 as moderate activity (Karnofsky Performance Score: 50–70), and group 3 as disabled individuals (Karnofsky Performance Score: 0–40). It was assumed that in the discriminant validity of PDS-II, patients with low Karnofsky scores acquired more demoralization scores.

##### Internal consistency

Cronbach α and Omega coefficients calculated internal consistency reliability of the scale in the 95% interval confidence.

### Statistics analyses

LISREL 8.8 for Windows (SSI Inc., Skokie, IL, USA) was used for evaluating the fitness indices and discriminant validities of PDS-II. To determine the factor structure of the PDS-II the one-factor model (Model II) and two-factor model (Model I) were assessed by evaluating the fitness indices. According to Khairi et al. (2021) the values of comparative fit index, incremental fit index, and non-normed fit index more than 0.90 indicated acceptable fit of model [31]. To determine the discriminant validity by using the structural equation model (SEM) in model I and model III, the AVE of the factors and the square correlation between the two factors in each model were examined.

Internal consistency reliability and convergent validity were assessed by using SPSS for Windows version 22 (SPSS Corporation, Chicago, IL, USA) plus extension. Kolmogorov–Smirnov (K-S) test was used to determine the normality of the PDS-II, GAD-7, PHQ-9, BHS and HADS. As all of the variables distributed abnormal according to K-S test (*P* < 0.05), so the Spearman rho test was used to determine the convergent validity of the PDS-II.

## Results

### Sample description

A total of 170 patients aged between 18 and 90 years (mean 55.61 years) suffering from cancer were evaluated in the study. Other demographic and clinical characteristics of the participants are shown in Table 1.

**Table 1:** Demographic characteristics of the participants (n = 170)

Variable		Frequency (%)	Mean (SD) of PDS-II score	Test
Demoralization Score	Total DS	-	11.8 (6.3)	-
	Meaning and Purpose	-	5.6 (3.1)	-
	Distress and Coping Ability	-	6.2 (3.7)	-
Age (year)	≥ 49	51 (30)	12.3 (6.7)	Kruskal Wallis *P* = 0.37
	50–60	55 (32.4)	11.0 (6.4)	
	≤ 61	64 (37.6)	11.1 (5.9)	
gender	Male	92 (54.1)	12.2 (6.0)	Mann-Whitney U *P* = 0.16
	Female	78 (45.9)	11.4 (6.6)	
Marital status	Never Married	14 (8.2)	12.9 (7.6)	Kruskal Wallis *P* = 0.63
	Married	144 (84.7)	11.7 (6.1)	
	Divorced/Widow/Separated	12 (7.1)	11.2 (6.7)	
education	Illiterate	16 (9.4)	10.6 (4.7)	Kruskal Wallis *P* = 0.29
	Primary School	96 (56.5)	11.0 (5.8)	
	Diploma	35 (20.6)	12.7(6.0)	
	University	23 (13.5)	14.4 (9.0)	
Job	Business Man	37 (21.8)	12.1(6.0)	Kruskal Wallis *P* = 0.20
	Employe	19 (11.2)	15.2 (8.4)	
	Workingman	33 (19.4)	12.0 (5.2)	
	Housekeeper	66 (38.8)	10.8 (6.2)	
	Workless	15 (8.8)	10.6 (5.3)	
Length of diagnosis (month)	1–12	117 (68.8)	11.1 (5.4)	Kruskal Wallis *P* = 0.15
	13–24	25 (14.7)	14.1 (8.0)	
	25–156	28 (16.5)	12.9 (7.5)	
Type of caner	Breast	19 (11.2)	8.5 (5.8)	Kruskal Wallis *P* = 0.05
	Blood	30 (17.6)	11.5 (7.5)	
	Colon	26 (15.3)	11 (4.2)	
	Lung	15 (8.8)	10.6 (4.8)	
	Gastric	45 (26.5)	11.8 (4.5)	
	Uterus And Ovaries	8 (4.7)	16.2 (8.8)	
	Other Types	27 (15.9)	14.3 (7.9)	
Stage of cancer	I	1 (0.6)	10.2 (-)	Kruskal Wallis without considering group I *P* = 0.02
	II	83 (48.8)	10.6 (5.6)	
	III	65 (38.2)	12.0 (5.7)	
	IV	21 (12.4)	15.8 (8.5)	

### Convergent validity

Convergent validity was examined using correlation coefficients of demoralization and its dimensions with GAD-7, PHQ-9, BHS, and HADS. As shown in Table 2, the score of GAD-7, PHQ-9, BHS, and HADS positively correlates with the total PDS-II score and its subscales. So the convergent validity of the PDS-II was confirmed.

**Table 2:** Convergent validity of the PDS-II using Spearman rho test

Variables	Mean (sd)	Demoralization Score	Mean-Purpose	Distress-Copping
GAD-7	14.61 (4.98)	r = 0.66 *p* < 0.001	r = 0.56 *p* < 0.001	r = 0.64 *p* < 0.001
PHQ	10.79 (5.66)	r = 0.65 *p* < 0.001	r = 0.63 *p* < 0.001	r = 0.56 *p* < 0.001
BHS	9.18 (4.1)	r = 0.46 *p* < 0.001	r = 0.52 *p* < 0.001	r = 0.34 *p* < 0.001
HADS	18.27 (6.42)	r = 0.41 *p* < 0.001	r = 0.45 *p* < 0.001	r = 0.31 *p* < 0.001

Note 1: DS-II: Demoralization Scale-Second Version; GAD-7: General Anxiety Disorder-7; PHQ: Patient Health Questionnaire; BHS: Beck’s Hopelessness Scale; HADS: Hospital Anxiety and Depression Scores

Note 2: Regard to the high correlation of DS-II and its dimensions with aligned tools, the convergent validity of DS-II was confirmed

### Construct validity

To determine the construct validity initially, CFA was used to assess two models. The overall model fit statistics are presented in Table 3. According to the values of fit indices (fit coefficients), both original 2-factor (model I) and one-factor (model II) models are acceptable.

**Table 3:** Goodness-of-fit indices of models of DS-II

Models	X^2^	df	X^2^/df	CFI	IFI	NFI	NNFI	SRMR	RMSEA
Model I^a^	247	103	2.39	0.93	0.93	0.88	0.91	0.073	0.091
Model II^b^	263	104	2.52	0.92	0.92	0.88	0.91	0.075	0.095
Model III^c^	597	272	2.2	0.93	0.94	0.89	0.93	0.072	0.084

Note. CFI = comparative fit index; df = degrees of freedom; IFI = incremental fit index; NFI = normed fit index; NNFI = non-normed fit index; RMSEA = root-mean-square error of approximation; SRMR = standardized root-mean-square residual; DS-II: Demoralization Scale-Second Version; PHQ: Patient Health Questionnaire

^a^As a Original 2-dimensional model and 16 items

^b^16 items of DS-II was evaluated as a one-dimensional model

^c^Two dimensional model including 16 items of DS-II as one factor, and 9 items of PHQ as another factor

The results showed that the AVE of purpose and meaning, and distress and coping ability were 0.31 and 0.37, respectively, which were less than the square correlation between these two dimensions (r^2^ = 0.79). It means that the correlation between the two subscales (purpose and meaning and distress and coping ability) is such high that discriminant validity between them wouldn’t be supported.

For determining the discriminant validity of PDS-II with depression, patients were divided into two groups with and without MDE. Table 4 shows the cross-tabulation frequencies of PHQ-9 and PDS-II in the groups. The frequency and percentage of the patients with a high, moderate, and low degree of demoralization in these groups were reported. According to Tables 4 and 95% of patients with high demoralization have a major depression, but it was only 13.9% among the patients with low demoralization.

**Table 4:** Cross-tabulation frequencies between demoralization and major depression

	PHQ N (%)		
Demoralization	No (10 >)	Yes (10 ≤)	Total
Low (0–7)	31 (86.1)	5 (13.9)	36 (100)
Moderate (7.1–15)	43 (47.3)	48 (52.7)	91 (100)
high (15.1≤)	2 (4.7)	41 (95.3)	43 (100)
Total	76 (44.7)	94 (55.3)	170 (100)

PHQ: Major Depressive Episodes; PDS-II: Persian version of Demoralization

Patients were divided into 3 categories according to PDS-II scores: low scorers (scores of 0–7;), moderate scorers (scores of 7.1–15), and high scorers (scores more than 15)

Patients were divided into 2 categories according to PHQ scores: Without Major Depressive Episodes (PHQ scores less than 10), and With Major Depressive Episodes (PHQ scores equals to or more than 10)

According to the Table 95.3% of those highly demoralized showed major depression

Also, the structural equation modeling showed that the AVE of the factors of DS and PHQ in model III were 0.32 and 0.42, respectively which were less than the square of the correlation between these two factors (r^2^ = 0.53). This shows that the correlation between the factors (PDS-II and PHQ-9) is such high that discriminant validity between them wouldn’t be supported.

In determining the discriminant validity of PDS-II according to participants’ function, the results showed that patients with a low Karnofsky score significantly acquired more demoralization (*p* < 0.001) (Table 5).

**Table 5:** Discriminant validity of PDS-II according to participants’ function

Karnofsky Performance Test	N	Mean (Sd)
Group 1 (Score: 80–100)	70	7.80 (3.90)
Group 2 (Score: 50–70)	88	13.82 (5.55)
Group 3 (Score: 0–40)	12	20.42 (6.93)

The average Karnofsky index score was 71

According to the one-way ANOVA test, there is a significant difference between Karnofsky’s performance scores in all groups (*p* < 0.001)

### Reliability

Cronbach α coefficients with 95% confidence intervals for the whole PDS-II and the meaning and purpose and distress and coping ability subscales 0.88 [CI: 0.83–0.91], 0.78 [CI: 0.69–0.83] and 0.82 [CI: 0.77–0.86], respectively. The omega coefficients for the whole PDS-II and its dimensions also were exactly the same coefficients.

## Discussion

The study aimed to determine the psychometric properties of the PDS-II in patients with cancer. In this regard, content validity, convergent validity, factor structure, discriminant validity, and internal consistency of the PDS-II were examined. In this study, the PDS-II and its dimensions demonstrated convergent validity with measures of psychological symptoms of general anxiety disorder, depression, hopelessness, and hospital anxiety and depression. Other studies also have revealed good convergent validity of DS-II with psychological symptoms [13, 15]. Compared with the distress and coping ability subscale, the meaning and purpose subscale yielded a stronger correlation with hospital anxiety and depression, and hopelessness. This observed difference suggests that loss of meaning and purpose has a more effect on hospital anxiety and depression, and hopelessness than a change in coping and distress. This is consistent with Robinson et al. study indicating that the meaning and purpose subscale showed more relationship with psychological symptoms [13].

Originally Robinson et al. (2016) introduced DS-II as a refined measure of demoralization consisting of 16 items and 2 subscales: meaning and purpose and distress and coping ability [16]. Koranyi et al. (2021) used Confirmatory Factor Analyses to evaluate whether the proposed two-factor solution of the original DS-II scale can be replicated with their sample of patients with cancer [15]. They tested two models: the one-factor model, aggregating across all 16 items, and the two-factor model according to the dimensional structure of two proposed subscales. Their results yielded similar borderline fit indices for both models, but considering their other results, the researchers suggested the one-factor model as an acceptable factor structure for DS-II. In this study, for assessing the factor structure of the PDS-II, confirmatory factor analysis was used for both two-dimensional and one-dimensional models of the scale. The results showed similar acceptable fit indices for both models. For a better conclusion about the superiority of one of the models, further investigation was performed to assess discriminant validity by using the structural equation modeling. The results showed that there is a high correlation between the subscales in the two-dimensional model in which the AVE of each factor (subscale) is less than the square of correlation coefficients between the subscales. So the subscales in the two-dimensional models are correlated highly and cannot be separated as an independent construct. Therefore, in this case, the discriminant validity of the two-dimensional PDS-II was rejected. Also, the acceptance of the one-dimensional model of the scale according to the fit indices is another proof that the discriminant validity of the PDS-II is rejected. This shows that 16 scale items are acceptable in the form of one dimension and cannot be separated into different dimensions.

In addition to the fact that the internal discriminant validity of the PDS-II has been challenged, there are also doubts about the external discriminant validity of the scale. The results showed that most of the participants with low demoralization and half of them with moderate demoralization have not shown major depression episodes, but 95% of the participants with high demoralization have shown major depression episodes. This matter is consistent with Robinson et al. [18] study that reported comorbidity between depression and demoralization exists at high levels of demoralization but not at moderate levels. On the other hand, comorbidity between high demoralization and major depression can make a question about the discriminant validity of PDS-II with depression. Because demoralized patients were significantly more likely to be depressed than those who were not classified as demoralized. It seems DS-24 that had overlaps with depression [32], DS-II has a lot of comorbidity with depression too. Also, the structural equation model showed that the correlation between the PDS-II and major depression (PHQ-9) is very high and they cannot be discriminated from each other. So although PDS-II has good external convergent validity, its external discriminant validity is not supported.

In analyzing discriminant validity in the groups with different Karnofsky’s performance scores, the results showed that patients with low Karnofsky index scores significantly acquired more demoralization scores. It means that the demoralization scale can differentiate the groups with low functional status from those who acquired higher scores in Karnofsky’s performance test. This matter might be due to the fact that one of the factors that may lead to demoralization in the patient refers to the feeling of the loss of independence [10].

For determining the internal consistency of the PDS-II, both the Cronbach alpha and omega coefficients were calculated and they were good for the whole scale and its dimensions. Previous studies have also confirmed the internal consistency of DS-II [14–16]. Although Robinson et al. (2016) acquired less alpha coefficient for the dimension of meaning and purpose in comparison with distress and coping ability, other studies have shown good alpha Cronbach’s coefficients for the meaning and purpose dimension [14–16]. Totally, PDS-II and its dimensions have good alpha and omega coefficients in our study that show there is a good agreement between the items in the whole scale and its dimensions, and the items might measure the same character.

### Limitation

This study had some limitations. First, this study used a convenient sample of patients with cancer whose results may not generalize to other clinical groups. Second, the researchers had to fill out the questionnaires through interviews with about 10% of the participants who were illiterate. Third, the stability of the scale has not been considered in the study. It is suggested to perform test-retest analyses in the next studies.

## Conclusion

According to the study, although the PDS-II has good content and convergent validity and good internal consistency among Iranian people with cancer, however, internal and external discriminant validity of the scale was not supported in the study. It is suggested to determine the discriminant validity of the DS-II in other populations. This study provides comprehensive information about the strengths and weaknesses of the PDS-II in diagnosing demoralization in patients with cancer and its differentiation from depression to researchers and healthcare providers.

## Data Availability

The dataset analysed during this study is available from the corresponding author on reasonable request.

## Abbreviations

DS-II: Demoralization Scale II
PHQ-9: Patient Health Questionnaire
BHS: Beck Hopelessness Scale
GAD-7: General Anxiety Disorder
HADS: Hospital Anxiety and Depression Scale
CVI: Content Validity Index
CFA: Confirmatory Factor Analyses
SEM: Structural Equation Model
AVE: Average Variance Extracted
MDE: Major Depression Episodes
CFI: Comparative Fit Index
DF: Degrees Of Freedom
IFI: Incremental Fit Index
NFI: Normed Fit Index
NNFI: Non-normed Fit Index
RMSEA: Root-Mean-Square Error of Approximation
SRMR: Standardized Root-Mean-square Residual
